# Simultaneous Multiparameter Mapping of the Liver in a Single Breath‐Hold or Respiratory‐Triggered Acquisition Using Multi‐Inversion Spin and Gradient Echo MRI

**DOI:** 10.1002/jmri.29584

**Published:** 2024-08-27

**Authors:** Mary Kate Manhard, Anandh Kilpattu Ramaniharan, Jean A. Tkach, Andrew T. Trout, Jonathan R. Dillman, Amol S. Pednekar

**Affiliations:** ^1^ Department of Radiology Cincinnati Children's Hospital Medical Center Cincinnati Ohio USA; ^2^ Department of Radiology University of Cincinnati College of Medicine Cincinnati Ohio USA

**Keywords:** rapid relaxometry, free breathing, liver, quantitative MRI

## Abstract

**Background:**

Quantitative parametric mapping is an increasingly important tool for noninvasive assessment of chronic liver disease. Conventional parametric mapping techniques require multiple breath‐held acquisitions and provide limited anatomic coverage.

**Purpose:**

To investigate a multi‐inversion spin and gradient echo (MI‐SAGE) technique for simultaneous estimation of T_1_, T_2_, and T_2_* of the liver.

**Study Type:**

Prospective.

**Subjects:**

Sixteen research participants, both adult and pediatric (age 17.5 ± 4.6 years, eight male), with and without known liver disease (seven asymptomatic healthy controls, two fibrotic liver disease, five steatotic liver disease, and two fibrotic and steatotic liver disease).

**Field Strength/Sequence:**

1.5 T, single breath‐hold and respiratory triggered MI‐SAGE, breath‐hold modified Look–Locker inversion recovery (MOLLI, T_1_ mapping), breath‐hold gradient and spin echo (GRASE, T_2_ mapping), and multiple gradient echo (mGRE, T_2_* mapping) sequences.

**Assessment:**

Agreement between hepatic T_1_, T_2_, and T_2_* estimated using MI‐SAGE and conventional parametric mapping sequences was evaluated. Repeatability and reproducibility of MI‐SAGE were evaluated using a same‐session acquisition and second‐session acquisition.

**Statistical Tests:**

Bland–Altman analysis with bias assessment and limits of agreement (LOA) and intraclass correlation coefficients (ICC).

**Results:**

Hepatic T_1_, T_2_, and T_2_* estimates obtained using the MI‐SAGE technique had mean biases of 72 (LOA: −22 to 166) msec, −3 (LOA: −10 to 5) msec, and 2 (LOA: −5 to 8) msec (single breath‐hold) and 36 (LOA: −43 to 120) msec, −3 (LOA: −17 to 11) msec, and 4 (LOA: −3 to 11) msec (respiratory triggered), respectively, in comparison to conventional acquisitions using MOLLI, GRASE, and mGRE. All MI‐SAGE estimates had strong repeatability and reproducibility (ICC > 0.72).

**Data Conclusion:**

Hepatic T_1_, T_2_, and T_2_* estimates obtained using an MI‐SAGE technique were comparable to conventional methods, although there was a 12%/6% for breath‐hold/respiratory triggered underestimation of T_1_ values compared to MOLLI. Both respiratory triggered and breath‐hold MI‐SAGE parameter maps demonstrated strong repeatability and reproducibility.

**Level of Evidence:**

1

**Technical Efficacy:**

Stage 2

Quantitative mapping of MR relaxation parameters (MR parametric mapping) is an important tool for the noninvasive detection, diagnosis, and evaluation of a wide range of liver pathologies.^1^, ^2^, ^3^ The MR relaxation parameters of solid organs can change in response to inflammation, fibrosis, fat, and iron deposition.^4^ Specific to the liver, T_1_ values have been reported to change with inflammation,^5^ fibrosis and cirrhosis,^6^ and steatosis.^7^ Hepatic T_2_ values have been reported to change in association with inflammation^8^ and the coexistent high water content of advanced fibrosis.^9^ T_2_* values in the liver have long been shown to accurately estimate hepatic iron concentration.^10^, ^11^ Through quantitative measurements, MR parameter mapping offers an objective and reproducible approach to assess hepatic disease which has the potential to improve disease diagnosis and staging.

While quantitative MRI measures offer valuable insights into abdominal organ properties, obtaining accurate MR parametric maps of large abdominal organs such as the liver requires long imaging times, multiple breath holds, and compensation for respiratory motion. Typically, hepatic MR parametric mapping is limited to quantifying a single parameter per scan, necessitating multiple scans using different sequences across multiple breath‐holds and with limited anatomic coverage to achieve a comprehensive multiparametric assessment.^12^, ^13^ Performing multiple consecutive breath‐holds while lying still in a scanner for a prolonged MRI examination is challenging, particularly for patients with respiratory compromise, obesity, other chronic conditions, and especially children. Furthermore, inconsistency in breath‐holds results in suboptimal image quality, repeated scans, extended scan times, and misalignment of anatomic positions across different parametric maps.

Recently, the multi‐inversion^14^, ^15^, ^16^ spin and gradient echo^17^, ^18^ (MI‐SAGE) method has been developed to efficiently acquire multiple MR contrasts across inversion and echo times in the brain,^19^ collecting a whole‐brain scan in 1–4 minutes. Multiple contrast images in MI‐SAGE enable simultaneous estimation of spatially aligned parametric maps, including T_1_, T_2_, and T_2_*.

Thus the aim of this study was to assess two versions of the MI‐SAGE method optimized for simultaneous estimation of hepatic T_1_, T_2_, and T_2_*: a single breath‐hold, 6 slice acquisition, and a respiratory triggered whole‐liver acquisition with a scan time of less than 1 minute. The MI‐SAGE estimated values were compared to commercially available, separate scans for T_1_, T_2_, and T_2_* mapping.

## Materials and Methods

### Participants

Participants with known chronic liver disease and asymptomatic healthy individuals were prospectively recruited under an IRB‐approved (IRB ID 2022‐0108), HIPAA‐compliant protocol with written informed consent obtained from all individuals. Healthy volunteers were recruited through hospital wide emails. Radiology department records were used to identify patients who had previously undergone MRI at our institution and been diagnosed with chronic liver disease. Inclusion criteria for the study included 1) being between ages 8–27 years old, 2) having the ability to lie still the MRI scanner, and 3) perform breath‐holds of at least 15 seconds. Exclusion criteria is absolute or relative contraindications of MRI. The study aimed to recruit approximately one‐third participants with hepatic steatosis (Proton Density Fat fraction (PDFF) >6% on prior MRI),^20^ one‐third with hepatic fibrosis from various underlying liver diseases (liver stiffness [LS] >3 kPa on prior MRI^21^), and one‐third healthy individuals.

### MI‐SAGE Acquisition Scheme

**Figure 1:**
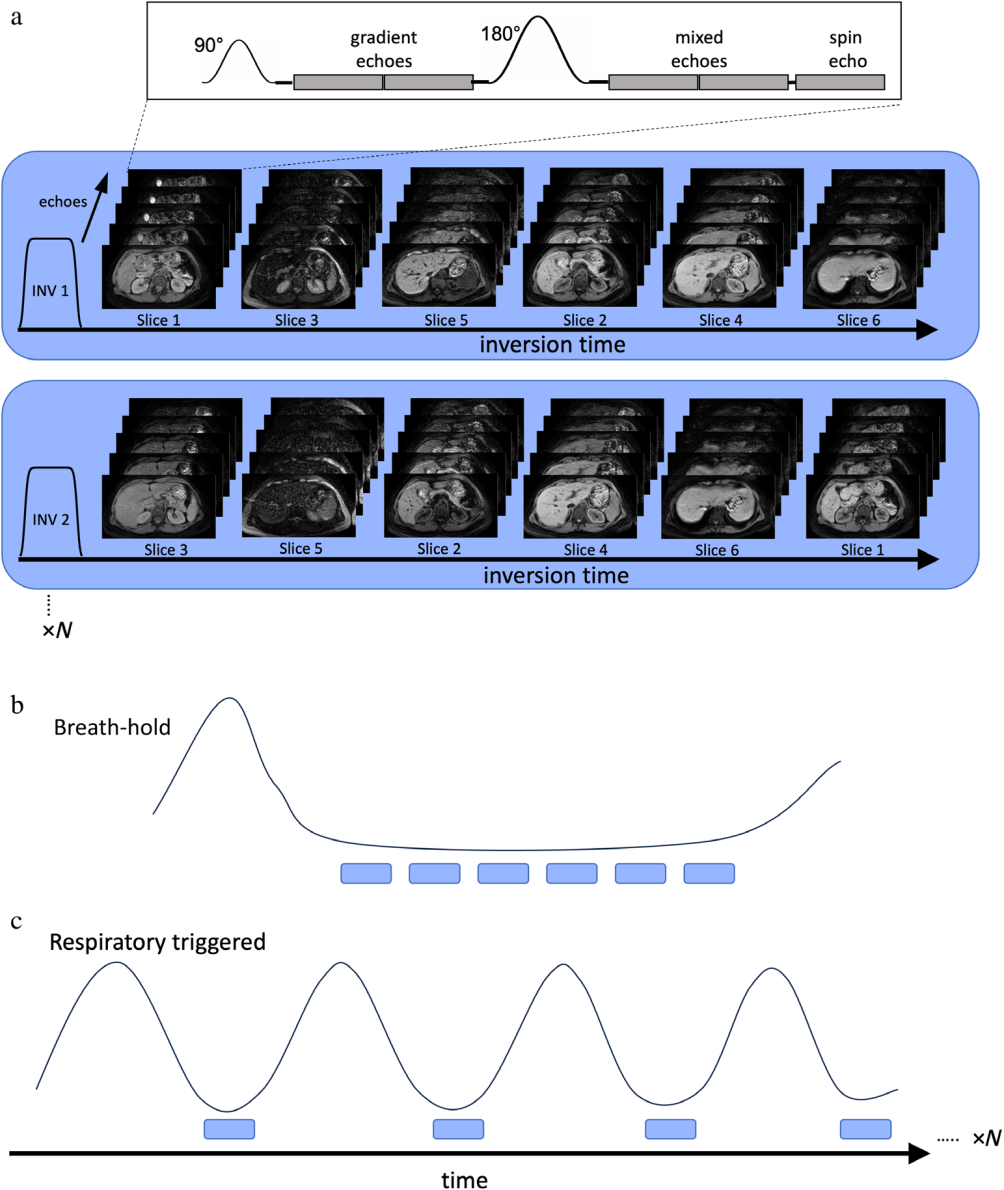
Schematic diagram illustrating the MI‐SAGE sequence for the liver. (**a**) The sequence uses a nonselective inversion pulse followed by the sequential acquisition of a prescribed number of slices S with incremental inversion times, this is repeated *N* (*N* = S/MB factor) times with a shifted slice ordering so that after *N* repetitions, all slices see the full range of *N* inversion times. For each slice, a single‐shot EPI SAGE acquisition is used to acquire gradient, mixed, and spin echoes. (**b**) A schematic of the respiratory bellows signal demonstrates that all *N* repetitions are acquired during a breath‐hold for bhMI‐SAGE. (**c**) The respiratory bellows signal and the triggering of the individual simultaneous multislice MI‐SAGE acquisition during exhalation is shown. The slice ordering is shifted after each respiratory period so that a different inversion delay time is experienced by each slice over *N* respiratory cycles. MB = multi band.

The MI‐SAGE method initially developed for brain^19^ was adapted for the abdomen as illustrated in Fig. 1a. In this method, a multiple inversion, interleaved multiple slice acquisition^14^, ^15^, ^16^ utilizes a nonselective adiabatic inversion pulse followed by the sequential acquisition of a prescribed number of slices (S) (or slice‐groups in the case of multiband [MB] acquisitions) through the abdomen. During each slice acquisition, a multiple spin‐and‐gradient echo (SAGE) sequence is utilized,^17^, ^18^ comprising a total of five echoes: two gradient echoes, two mixed spin‐and‐gradient echoes, and a spin echo. A fat‐suppressed single‐shot echo planar imaging (EPI) read out enables rapid acquisition of each echo. These five echoes enable the estimation of both T_2_ and T_2_* maps for each slice. The MI‐SAGE acquisition is repeated *N* times, where *N* = S/MB factor, with a shifted slice ordering for each repetition. This allows acquisition of each slice over a range of inversion times suitable for estimating target range of T_1_ values.

The MI‐SAGE acquisition scheme was adapted to allow either a single breath‐hold (Fig. 1b) or a respiratory‐triggered (Fig. 1c) acquisition. In breath‐hold acquisitions the repetition time (TR) between inversion pulses and number of repetitions (*N*) are chosen to achieve a balance between sufficient T_1_ recovery (>3× longest target normal T_1_) and a sustainable breath‐hold time. In the respiratory triggered acquisitions, signal from a respiratory bellows is used to detect the onset of expiration to trigger the application of the inversion pulse followed by SAGE acquisition of S slices while the patient breathed normally. This is followed by a pause until the subsequent expiratory trigger point, where the slice order for the MI‐SAGE acquisition is shifted, continuing until *N* repetitions are acquired, resulting in *N* TIs per slice.

### Protocol

All imaging was performed on a 1.5 T scanner (Ingenia, Philips Healthcare, Best, The Netherlands) using a torso coil with a combination of 16 anterior and 12 posterior elements. Detailed imaging parameters for all sequences are provided in Table 1. Briefly, the breath‐hold MI‐SAGE (bhMI‐SAGE) protocol prescribed six slices through the liver with in‐plane acceleration of three and no multiband (MB factor = 1). This scan utilized six repetitions resulting in six different inversion times for each slice, in addition to two starting dummy repetitions to achieve steady state. The TR was set to 2 seconds, to allow adequate T_1_ recovery time for the liver at 1.5 T. The total breath‐hold duration was 16 seconds. The respiratory‐triggered MI‐SAGE (rtMI‐SAGE) protocol prescribed a total of 20 slices across the whole liver. Within each respiratory cycle, the inversion pulse was followed by the SAGE acquisition, with a minimum gap of 3 seconds between two expiratory triggers. The rtMI‐SAGE used 10 repetitions plus two starting dummy repetitions, resulting in 10 TIs per slice, with an in‐plane acceleration of three, and MB factor of two. The respiratory bellows signal was logged in real‐time directly on the scanner. Both MI‐SAGE protocols were repeated once to evaluate repeatability, and again after taking the participant out of the scanner and repositioning to evaluate reproducibility. Parametric maps based on MI‐SAGE were computed offline using a dictionary matching approach, described in the following text. Fat suppressed hepatic T_1_, T_2_, and T_2_* maps were also generated by the scanner from a single mid‐liver slice location with the same field of view and nominally identical acquired spatial resolution using commercially available sequences. For T_1_ mapping, a modified Look–Locker inversion recovery (MOLLI)^11^ was implemented with a 5 s(3 s)3 s acquisition scheme, resulting in eight total TIs. The gradient and spin echo (GRASE) T_2_ mapping sequence, initially validated for myocardial T_2_ estimation in a breath‐hold,^22^ has also been adapted for abdominal organ imaging^23^, ^24^ and was used as the comparison method in this study. A multiple gradient echo (mGRE) sequence was used for quantification of T_2_* in a single breath‐hold.^25^ All mapping sequences were acquired with fat suppression using a SPIR fat saturation pulse.

**Table 1:** Scan Parameters

	bhMI‐SAGE	Rt MI‐SAGE	MOLLI	GRASE	mGRE
FOV (mm)	440 × 440	440 × 440	440 × 440	440 × 440	440 × 440
Slice thickness (mm)	10	10	10	10	10
No. slices	6	20	1	1	1
Resolution (mm)	3 × 3	3 × 3	3 × 3	3 × 3	3 × 3
Accel. factor	SENSE = 3, MB = 1	SENSE = 3, MB = 2	SENSE = 2	SENSE = 2	SENSE = 1
TR (msec)	2000	1 respiratory cycle (minimum 3000)	1.9	1000	1000
TE (msec)	[10/34/71/95/119]	[11/39/83/111/139]	0.8	15	6
No. echoes/echo spacing (msec)	5/variable	5/variable	1/−	9/7.4	16/2
Flip angle (°)	90	90	35	90	25
No. TIs/First TI/TI spacing (msec)	6/75/149	10/70/149	8/47/198	–	–
Fat sat	SPIR	SPIR	SPIR	SPIR	SPIR
Duration (seconds)	16	10 respiratory cycles	11	14	15
Map(s)	T_1_/T_2_/T_2_*	T_1_/T_2_/T_2_*	T_1_	T_2_	T_2_*

bhMI‐SAGE = breath‐hold multiple inversion spin and gradient echo; rtMI‐SAGE = respiratory‐triggered multiple inversion spin and gradient echo; MOLLI = modified Look‐Locker; GRASE = gradient and spin echo; mGRE = multiple gradient echo; SENSE = sensitivity encoding; MB = multiband; SPIR = spectral presaturation with inversion recovery.

### Estimation of Parameters Using MI‐SAGE

Parametric maps based on MI‐SAGE were generated offline using MATLAB (version R2019b; The MathWorks Inc., Natick, MA, USA). The signal of each voxel across echo time and inversion time were fitted and matched within a dictionary of the signal evolutions computed using Bloch simulations. The dictionary was computed for combinations of T_1_, T_2_, and T_2_* values in ranges expected in the abdomen (100–2000 msec T_1_, 10–600 msec T_2_, and 10–600 msec T_2_*, starting with the first value in the range and incrementing in steps of 5% until the maximum value in the range was reached). For respiratory triggered acquisitions, dictionaries were computed based on the participants' recorded respiratory signal and acquisition time intervals. For breath‐hold acquisitions, the dictionary was computed once for all subjects. Dictionary matching was performed for each voxel over the 30 (bhMI‐SAGE) or 50 (rtMI‐SAGE) source images to generate estimated T_1_, T_2_, and T_2_* maps.

### Data Analysis

In all participants, a freehand region of interest (ROI) with a minimum size of 100 mm^2^ was manually drawn by a research associate with 2 years of experience in abdominal MRI research (A.K.R.) in the right hepatic lobe at an approximately matching anatomic location on all of the parametric maps. Care was taken to avoid any visible blood vessels, parenchyma edge, bile ducts, and the liver capsule. The mean and standard deviation of the values within the ROIs were recorded for each mapping method.

### Statistical Analysis

Descriptive statistics including mean, standard deviation, and range were reported across all participants' T_1_, T_2_, and T_2_* ROI values for all methods. When reporting participant's mean MI‐SAGE values, the median of the means of three repeated scans was used. Bland–Altman analysis and intraclass correlation coefficients (ICCs) were used to evaluate absolute agreement between MI‐SAGE and conventional methods, between bhMI‐SAGE and rt‐MISAGE, as well as between repeated MI‐SAGE measures. A *P*‐value <0.05 was considered significant for all inference testing. The mean bias was reported as the difference between two measures in milliseconds, and 95% limits of agreement (LOA) were calculated as appropriate. Correlation coefficients were interpreted as follows: 0–0.19, very weak; 0.2–0.39, weak; 0.40–0.59, moderate; 0.60–0.79, strong; and 0.80–1.0, very strong.^26^ Statistical analyses were performed using MATLAB and GraphPad Prism (v10.1.1, GraphPad Software, LLC).

## Results

Twenty‐one study participants were recruited and underwent an MRI examination. Five participants had incomplete parametric maps due to either limited image quality (*N* = 2) or MRI conditional implants which prevented the use of RF intensive MB for rtMI‐SAGE (*N* = 3) and were thus excluded from the analysis. The remaining 16 subjects included in the analysis ranged from 9 to 25 years old (eight male) and consisted of healthy controls (*N* = 7), participants with steatotic liver disease (*N* = 5), participants with hepatic fibrosis (*N* = 2), and participants with both steatotic liver disease and hepatic fibrosis (*N* = 2). The mean fat fraction was 18.8 ± 12.4 (2.7–32)% in participants with steatotic liver disease. The mean LS value was 3.9 ± 2.7 (2.1–10.24) kPa in participants with hepatic fibrosis. Participant demographics are summarized in Table 2. For the MI‐SAGE map estimation, the dictionary generation took approximately 2–3 minutes per slice and dictionary matching took an additional 2–3 minutes per slice, in our offline implementation.

**Table 2:** Demographic Characteristics of Study Participants

Number of patients	16
Age (years)	17.5 ± 4.6 (9–25)
Pediatric:Adult	8:8
Female:Male	8:8
Height (cm)	166.5 ± 10.9 (142–188)
Weight (kg)	78.3 ± 35.2 (38.6–165.3)
BMI (kg/m^2^)	27.6 ± 10.1 (16.2–51.9)
Clinical indications	
Healthy	7
Steatotic liver disease	5
Fibrotic liver disease	2
Steatotic and fibrotic liver disease	2

Values are counts or mean with standard deviations with ranges presented in parentheses. Steatotic liver disease defined as PDFF >6%, Fibrotic liver disease defined as stiffness >3 kPa, pediatric = age < 18.

BMI = body mass index.

**Figure 2:**
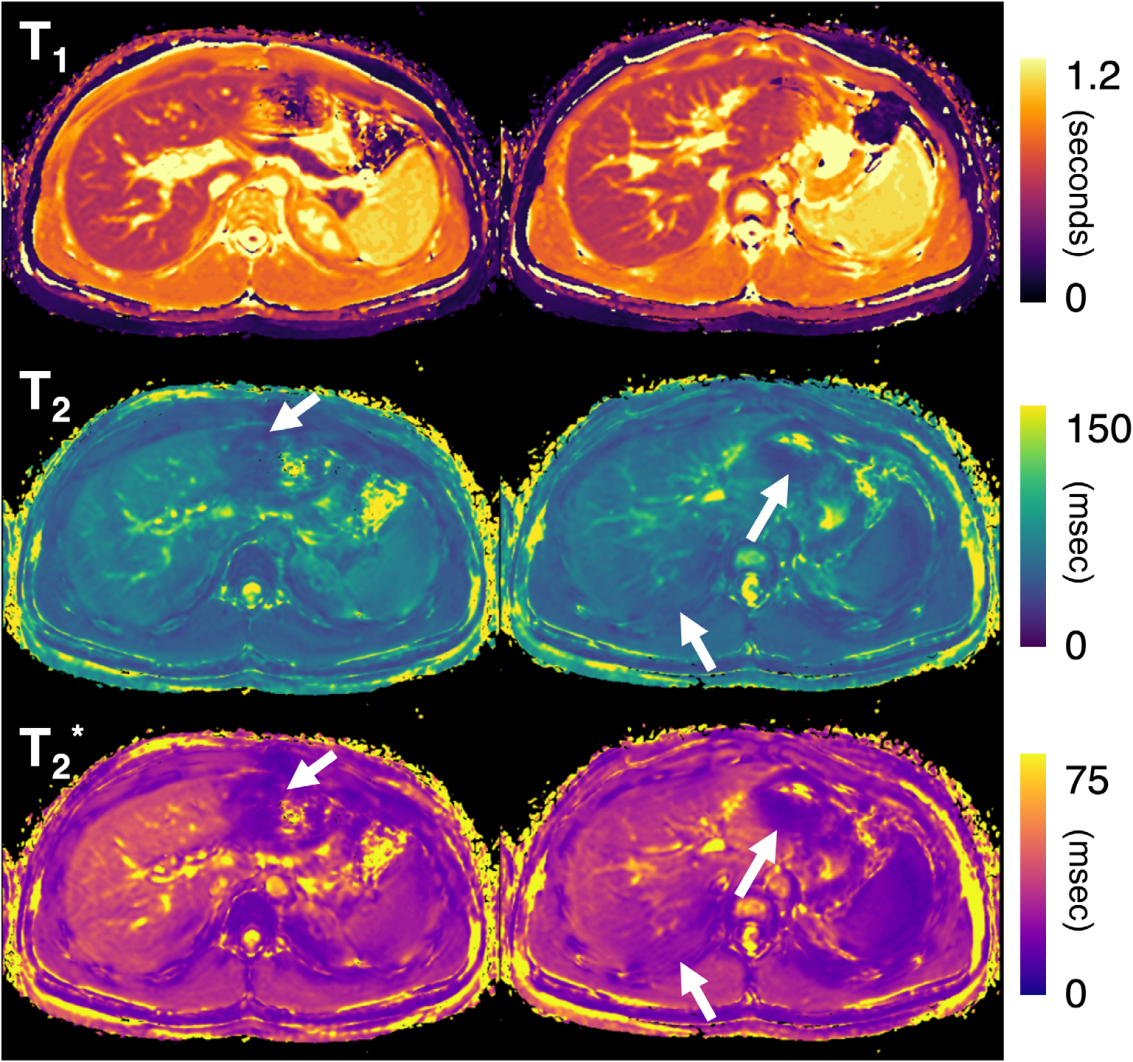
A 16‐year‐old male participant with hepatic fibrosis. Representative T_1_ maps (top), T_2_ maps (middle), and T_2_* maps (bottom) estimated from the rtMI‐SAGE acquisition for two slices at the portal hilum level. Field inhomogeneity‐related signal drop is noted in T_2_ and T_2_* maps (arrows).

**Figure 3:**
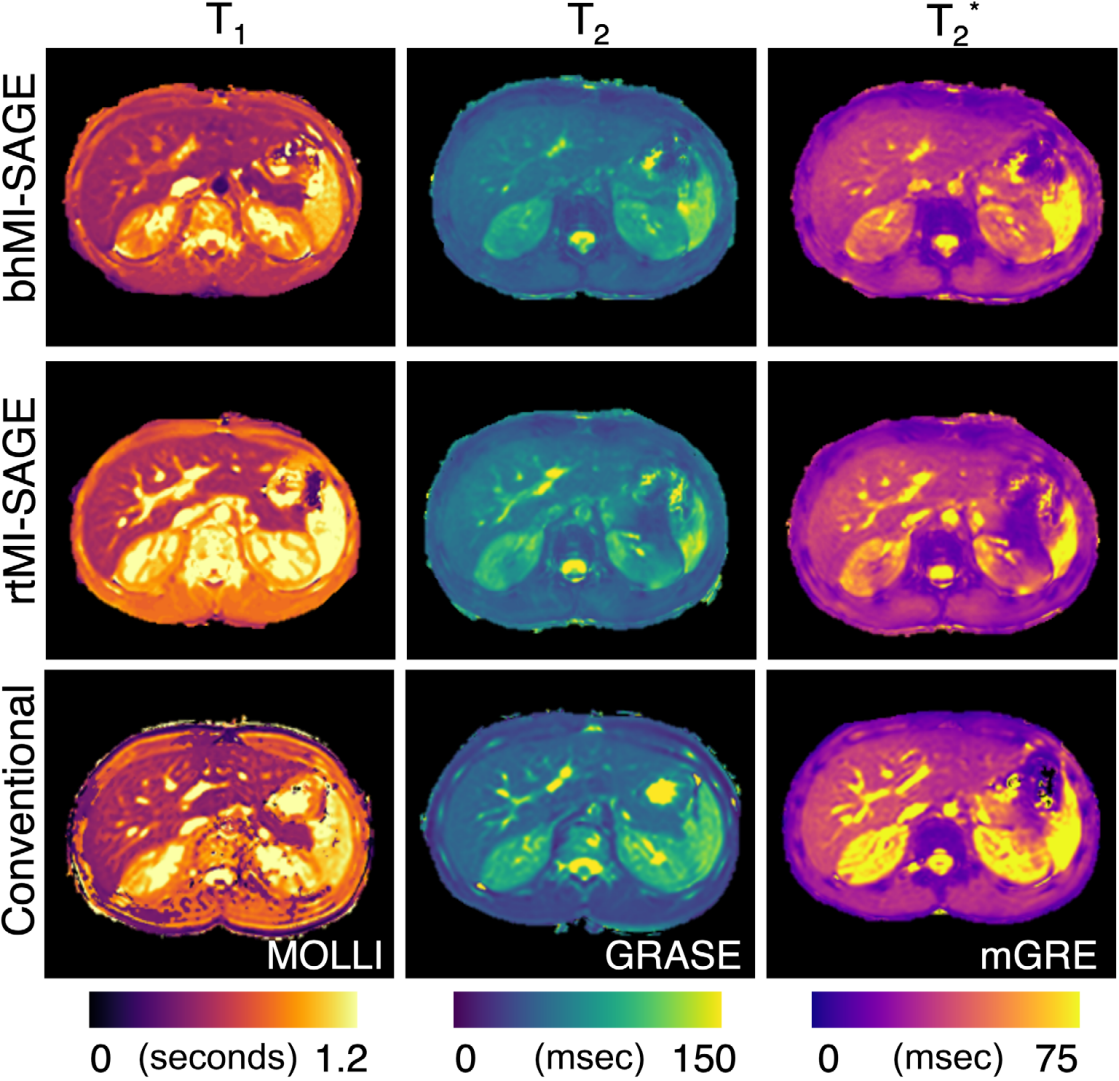
A 13‐year‐old male healthy participant. Representative T_1_, T_2_, and T_2_* maps obtained using bhMI‐SAGE (top), rtMI‐SAGE (middle), and commercially available breath‐hold mapping techniques (bottom).

The average hepatic T_1_ value was 533 ± 25, 563 ± 40, and 599 ± 64 msec with bhMI‐SAGE, rtMI‐SAGE, and MOLLI, respectively. Average hepatic T_2_ value was 55 ± 8, 56 ± 11, and 52 ± 7 msec with bhMI‐SAGE, rtMI‐SAGE, and GRASE. T_2_* values obtained with bhMI‐SAGE, rtMI‐SAGE, and mGRE was 34 ± 7 msec, 32 ± 8 msec, 36 ± 6 msec on average, respectively (Table 3, Figs. 2 and 3). bhMI‐SAGE showed very strong agreement with conventional methods for T_2_ and T_2_* (ICC: 0.87–0.88) and a moderate agreement for T_1_ (ICC: 0.52), while rtMI‐SAGE showed strong agreement for T_1_ and T_2_ (ICC: 0.65–0.68) while very strong agreement was observed for T_2_* (ICC: 0.87) (Table 4).

**Table 3:** Relaxometry Values From MI‐SAGE and Conventional Methods

	T_1_ (msec)	T_2_ (msec)	T_2_* (msec)
All participants (*N* = 16)			
bhMI‐SAGE	533 ± 25 (471–571)	55 ± 8 (45–72)	34 ± 7 (25–48)
rtMI‐SAGE	563 ± 40 (505–671)	56 ± 10 (40–75)	32 ± 8 (21–49)
Conventional methods	599 ± 64 (486–702)	52 ± 7 (42–69)	36 ± 6 (27–45)
Healthy volunteers (*N* = 7)			
bhMI‐SAGE	535 ± 23 (512–571)	60 ± 9 (46–72)	38 ± 7 (26–48)
rtMI‐SAGE	559 ± 55 (505–671)	59 ± 11 (40–75)	35 ± 9 (21–49)
Conventional methods	591 ± 64 (514–702)	56 ± 8 (48–69)	39 ± 5 (32–45)
Steatotic liver disease (*N* = 5)			
bhMI‐SAGE	528 ± 37 (471–564)	50 ± 5 (45–58)	29 ± 4 (26–36)
rtMI‐SAGE	559 ± 32 (531–596)	52 ± 10 (44–68)	26 ± 4 (22–31)
Conventional methods	588 ± 81 (486–671)	48 ± 5 (42–54)	31 ± 4 (27–37)
Hepatic fibrosis (*N* = 2)			
bhMI‐SAGE	524 ± 1 (523–525)	59 ± 1 (58–59)	38 ± 3 (36–39)
rtMI‐SAGE	565 ± 7 (560–570)	62 ± 3 (60–64)	35 ± 4 (32–37)
Conventional methods	600 ± 9 (594–607)	54 ± 4 (51–57)	40 ± 0 (40–40)
Steatotic + fibrosis (*N* = 2)			
bhMI‐SAGE	548 ± 1 (547–548)	51 ± 8 (45–57)	31 ± 8 (25–37)
rtMI‐SAGE	586 ± 12 (578–595)	52 ± 10 (45–59)	29 ± 10 (22–35)
Conventional methods	651 ± 60 (609–693)	47 ± 5 (44–50)	31 ± 5 (27–34)

Values are mean ± standard deviation (range). Conventional methods indicate MOLLI for T_1_, GRASE for T_2_, and mGRE for T_2_*.

bhMI‐SAGE = breath‐hold multiple inversion spin and gradient echo; rtMI‐SAGE = respiratory‐triggered multiple inversion spin and gradient echo.

**Table 4:** Repeatability, Reproducibility, and Agreement of MI‐SAGE Sequences With Conventional Methods

		Compared to Conventional		Repeatability		Reproducibility	
		bhMI‐SAGE	rtMI‐SAGE	bhMI‐SAGE	rtMI‐SAGE	bhMI‐SAGE	rtMI‐SAGE
T_1_	Bias	72	36	‐6	4	−11	−5
	(LOA)	(−22 to 166)	(−43 to 120)	(−42 to 30)	(−51 to 59)	(−56 to 35)	(−55 to 46)
	ICC	0.52	0.68	0.77	0.77	0.72	0.82
	(CI)	(0.06–0.80)	(0.29–0.87)	(0.45–0.91)	(0.45–0.91)	(0.36–0.89)	(0.55–0.93)
T_2_	Bias	−3	−3	−1	0	−1	−2
	(LOA)	(−10 to 5)	(−17 to 11)	(−6 to 3)	(−8 to 8)	(−9 to 6)	(−14 to 11)
	ICC	0.88	0.65	0.96	0.90	0.91	0.92
	(CI)	(0.69–0.96)	(0.25–0.86)	(0.89–0.99)	(0.75–0.97)	(0.76–0.97)	(0.78–0.97)
T_2_*	Bias	2	4	−1	0	−1	−1
	(LOA)	(−5 to 8)	(−3 to 11)	(−7 to 5)	(−5 to 6)	(−8 to 7)	(−8 to 5)
	ICC	0.87	0.87	0.90	0.94	0.88	0.92
	(CI)	(0.66–0.95)	(0.66–0.95)	(0.74–0.96)	(0.84–0.98)	(0.69–0.96)	(0.78–0.97)

Bias and limits of agreement (LOA) are reported in milliseconds. ICC values are presented with 95% confidence intervals (CIs) in parentheses. Conventional methods indicate MOLLI for T_1_, GRASE for T_2_, and mGRE for T_2_*.

bhMI‐SAGE = breath‐hold multiple inversion spin and gradient echo; rtMI‐SAGE = respiratory‐triggered multiple inversion spin and gradient echo.

**Figure 4:**
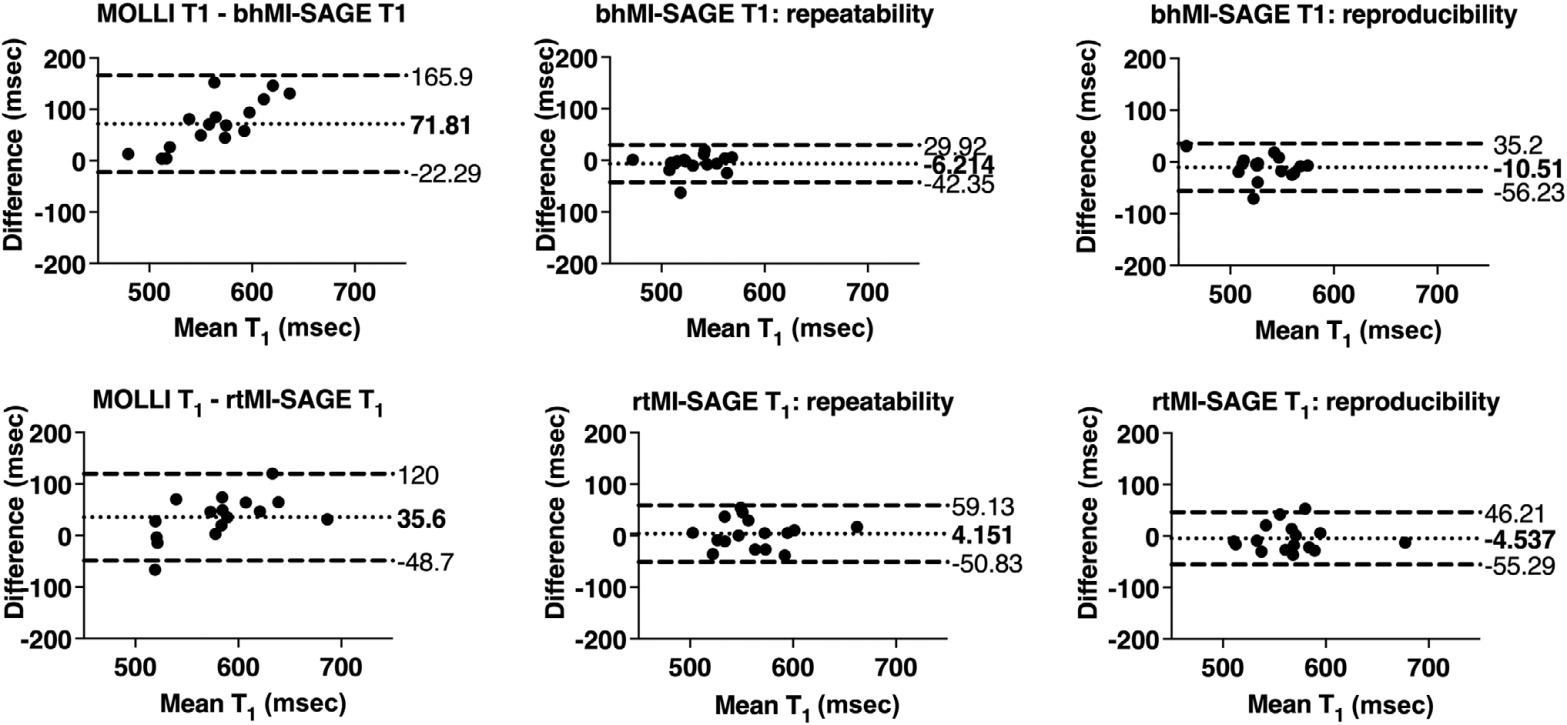
Bland–Altman plots of T_1_ estimates obtained using bhMI‐SAGE (top) and rtMI‐SAGE (bottom) for all participants. Values obtained with MI‐SAGE are compared to those obtained by fat suppressed MOLLI (left), repeated MI‐SAGE showing repeatability (middle), and repeated MI‐SAGE after repositioning showing reproducibility (right).

The bhMI‐SAGE underestimated T_1_ values with a mean bias of 72 msec or 12.3% (95% LOA: −22 to 166 msec) and rtMI‐SAGE underestimated T_1_ values with a bias of 36 msec or 5.8% (95% LOA: −49 – 120 msec) compared to the T_1_ values estimated with fat suppressed MOLLI (Table 4, Fig. 4). Both bhMI‐SAGE and rtMI‐SAGE showed strong T_1_ repeatability and reproducibility with little bias (Table 4, Fig. 4).

**Figure 5:**
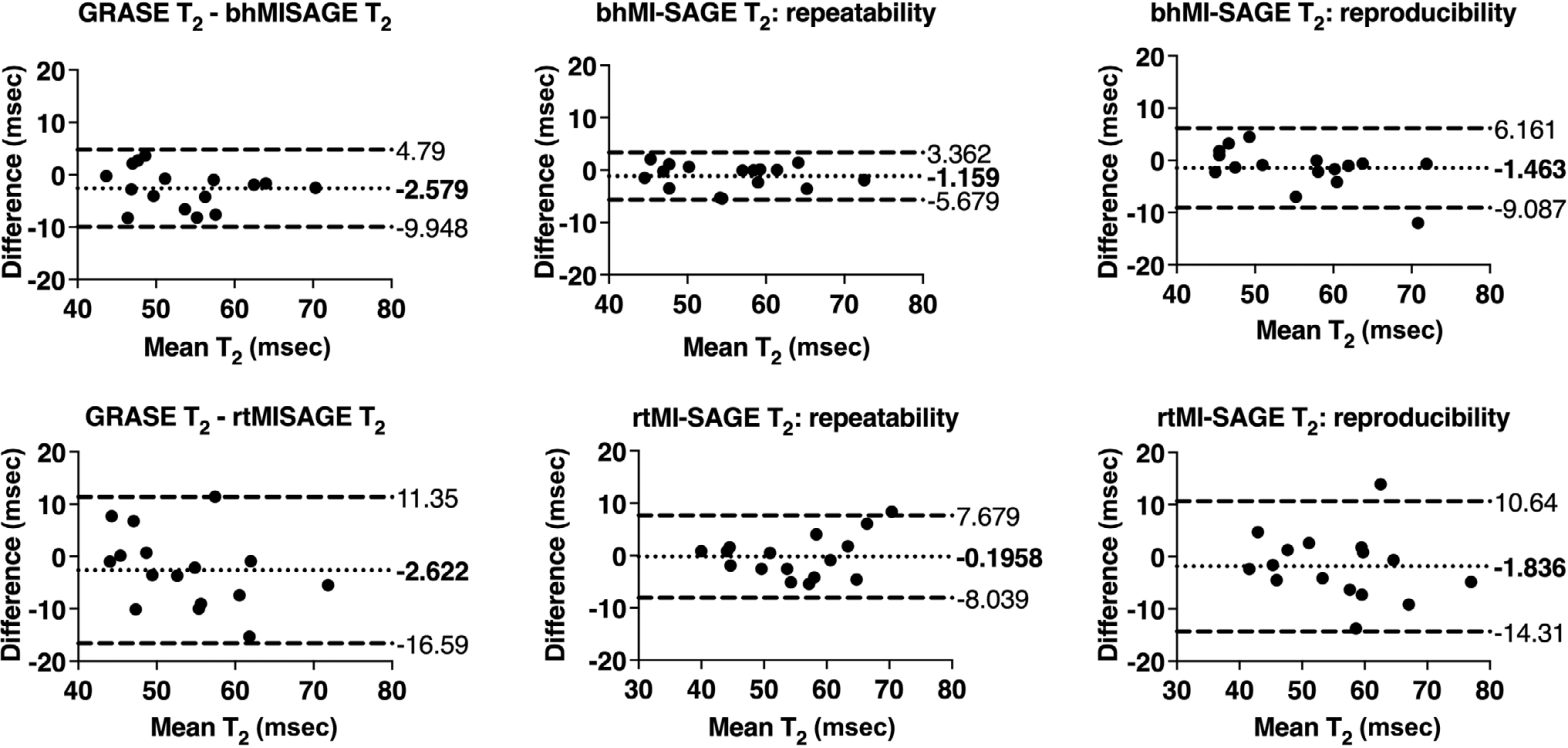
Bland–Altman plots of T_2_ estimates obtained using bhMI‐SAGE (top) and rtMI‐SAGE (bottom) for all participants. Values obtained with MI‐SAGE are compared to those obtained with fat suppressed GRASE (left), repeated MI‐SAGE showing repeatability (middle), and repeated MI‐SAGE after repositioning showing reproducibility (right).

**Figure 6:**
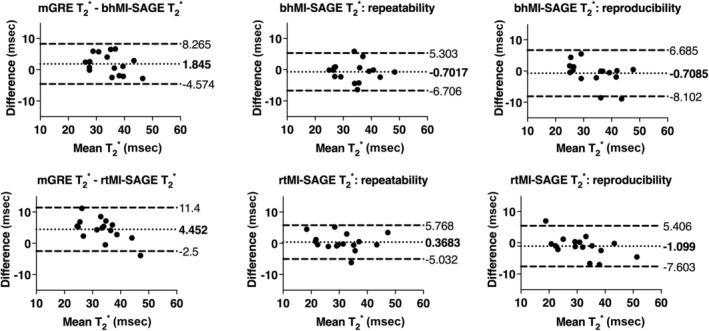
Bland–Altman plots of T_2_* estimates obtained using bhMI‐SAGE (top) and rtMI‐SAGE (bottom) for all participants. Values obtained with MI‐SAGE are compared to fat suppressed mGRE (left), repeated MI‐SAGE showing repeatability (middle), and repeated MI‐SAGE after repositioning showing reproducibility (right).

Biases of T_2_ compared to fat suppressed GRASE were as follows: −2.6 msec or −4.7% (95% LOA: −10 to 5 msec) and −2.6 msec or −4.3% (95% LOA: −17 to 11 msec) for bhMI‐SAGE and rtMI‐SAGE, respectively (Table 4, Fig. 5). T_2_* biases compared to fat suppressed mGRE were 1.8 msec or 5.9% (95% LOA: −5 to 8 msec) for bhMI‐SAGE and 4.4 msec or 15.0% (95% LOA: −3 to 11 msec) for rtMI‐SAGE (Fig. 6). Both T_2_ and T_2_* obtained using MI‐SAGE had very strong repeatability and reproducibility for bhMI‐SAGE (ICC: 0.88–0.96) and rtMI‐SAGE (ICC: 0.90–0.94) (Table 4). When comparing rtMI‐SAGE and bhMI‐SAGE, there was a mean bias of 36 msec (95% LOA: −38 to 110 msec) in T_1_ values, −0.04 msec (95% LOA −11 to 11 msec) in T_2_ values, and −2.6 msec (95% LOA: −10 to 4 msec) in T_2_* values.

## Discussion

Estimated hepatic T_1_, T_2_, and T_2_* values obtained simultaneously using an MI‐SAGE sequence optimized for the liver did not differ significantly from those obtained using conventional individual parametric mapping sequences. A breath‐hold version of MI‐SAGE provided all parametric maps in six slices in a 16‐second breath‐hold, while the respiratory triggered version provided all parametric maps in 20 slices across the entire liver in less than a minute. Quantitative MI‐SAGE maps estimated over a moderate but clinically relevant range of T_1_, T_2_, and T_2_* values exhibited strong to very strong repeatability and reproducibility.

Several approaches have been proposed for simultaneous multiparametric mapping in the liver. MR fingerprinting (MRF) has been used to estimate liver T_1_, T_2_, and T_2_* and PDFF^27^ in one breath‐hold per slice. Additionally, a free‐breathing 2–4 minutes scan time MRF protocol has been used to estimate T_1_ and T_2_ in the pancreas.^28^ Another method, MR multitasking, uses multiecho acquisitions for T_1_, PDFF, and T_2_* quantification across the entire liver in a 5‐minute free‐breathing acquisition.^29^ While promising, these methods still face limitations in terms of either anatomic coverage, scan duration, or achievable number of relaxometry parameters. These alternative techniques also typically acquire hundreds of highly undersampled image contrasts, where each individual contrast image may not be of diagnostic quality but together can be robustly fit to a dictionary of signal evolutions to estimate parametric values. Conversely, MI‐SAGE acquires much fewer contrasts (~30–50), each with diagnostic image quality and uses the remaining time to spatially encode data. This methodology results in increased anatomic coverage and a relatively motion‐robust scan, as each image is obtained using a single‐shot acquisition. The MI‐SAGE techniques presented here were able to achieve simultaneous T_1_, T_2_, and T_2_* maps with full‐liver coverage in either a single breath‐hold scan, or a single respiratory triggered scan.

While MOLLI^14^ has increasingly been used to estimate T_1_ in abdominal organs^30^ with relatively high reproducibility,^31^ the method requires an 11‐second breath‐hold for each imaging slice, making complete liver coverage impractical. The MI‐SAGE technique allows for T_1_ mapping of the entire liver, and uses slice shifting to efficiently sample the inversion recovery curve. Both breath‐hold and respiratory triggered MI‐SAGE underestimated T_1_ compared to fat suppressed MOLLI, but this underestimation did not reach statistical significance. While the presence of fat in the tissue is known to confound T_1_ estimates,^32^, ^33^ in this study, all the T_1_ mapping acquisitions used the identical spectral inversion recovery (SPIR) based fat suppression to mitigate confounding effects of fat on T_1_ estimates. Furthermore, the fat suppressed MOLLI protocol acquired the center of k‐space first to allow more effective fat suppression. Therefore, this observed bias most likely stems from a combination of other factors, such as differences among sequences in terms of sampling scheme of inversion times and total inversion recovery time.

The underestimation bias (relative to MOLLI) with rtMI‐SAGE was less than that with bhMI‐SAGE. This may be attributed to the longer signal recovery time between successive inversion pulses in rtMI‐SAGE (minimum 3 seconds) compared to that in bhMI‐SAGE (2 seconds), while for MOLLI this time was 8 seconds. In the case of the bhMI‐SAGE acquisition, the TR was constrained to 2 seconds to keep the breath‐hold to a feasible duration (16 seconds), which greatly shortened the amount of signal recovery between inversion pulses and limited the longest sampling point after the inversion compared to the MOLLI acquisition scheme of 5 s(3 s)3 s. Although this constraint was removed in the rtMI‐SAGE acquisition, the signal recovery period varied depending upon participants respiratory patterns. While this variation was accounted for in the Bloch simulations by using the participant's respiratory cycle times during the acquisition, it may have resulted in diminished predictability and a more difficult pattern matching for the dictionary. Additionally, misalignment of images due to inconsistent respiratory depth could lead to deviation in signal evolution compared to Bloch simulations, which can affect T_1_ estimation through dictionary matching. Magnetization transfer effects of the SPIR fat suppression pulse can also influence the accuracy of T_1_ in multislice acquisitions, which may have contributed to the underestimation bias seen in the MI‐SAGE versus the single‐slice MOLLI acquisition.

While GRASE T_2_ mapping and mGRE T_2_* mapping are well validated parametric mapping methods, they still require separate individual scans and separate breath‐holds. Additionally, the utility of GRASE T_2_ mapping remains constrained as it can only acquire 1–2 slices per breath‐hold, resulting in partial coverage of abdominal organs. Both T_2_ and T_2_* MI‐SAGE maps were acquired in a single breath‐hold or respiratory‐triggered scan, with multiple slices covered. The T_2_ and T_2_* MI‐SAGE values showed very good agreement with the conventional methods. However, outside of the hepatic ROI that was used for analysis, artifacts and signal dropouts were noted in the MI‐SAGE maps of these parameters in some cases which can be attributed to the single‐shot EPI readout of the MI‐SAGE acquisition. While the EPI‐based MI‐SAGE allows for the time efficient acquisition of many contrasts, it is highly susceptible to field inhomogeneities observed in abdominal regions, particularly near air–tissue interfaces.^34^ EPI sequences also necessitate robust and uniform fat suppression to eliminate artifacts and ghosting, which is challenging to achieve in large field of view abdominal scans in the presence of visceral and subcutaneous fat.^35^, ^36^ Using goodness of fit based confidence maps can help to guide ROI placement into regions where the dictionary matching performed well. However, challenges of EPI need to be considered in future studies, including further optimization of sequence parameters, or employing alternative fast readouts to overcome the present limitations and improve images and maps.

Free‐breathing and breath‐hold MI‐SAGE scans each have their advantages depending on the specific clinical situation, the patient's ability to cooperate, and the imaging goals. Breath‐hold scans are typically most efficient and more robust to motion and may be the most practical option when the patient is compliant and minimizing scan time is important. However, free‐breathing scans are more comfortable for patients and can reduce motion artifacts that may arise from improper/failed breath‐holds. For quantitative mapping, free‐breathing protocols relax the time constraint of a feasible breath‐hold duration, allowing for more sampling points for more accurate estimation and increased slice coverage. The rtMI‐SAGE technique allows the patient to breath normally, and also allows for longer T_1_ recovery time between slice acquisitions, which can improve the accuracy of T_1_ maps, as inferred from the reduced T_1_ bias in this study with rtMI‐SAGE compared to bhMI‐SAGE.

### Limitations

This study was limited by the inclusion of only 16 relatively young research participants, each of whom was capable of performing consistent 16‐second breath‐holds. Further, while we compared the parametric maps from MI‐SAGE directly to conventional parametric maps, liver biopsies were not performed and were thus not available as a reference. By design, a mixture of participants with healthy liver and with chronic liver disease were recruited, although the study population with high liver fat fraction or fibrosis was small, making it difficult to understand specific differences in parametric maps due to disease in this cohort. Using conventional mapping sequences, only a single slice was acquired due to time constraints, so only single hepatic ROI analysis was performed by a single reader with no intra‐ or inter‐reader reproducibility assessed. Future studies with larger sample sizes, more clinical diagnostic markers of disease, and multiple readers will allow for a more thorough investigation into how these relaxometry values may help in diagnoses or monitoring disease progression. These studies will also help determine the allowable limits of repeatability and reproducibility needed from the MI‐SAGE method.

## Conclusion

Quantitative parametric maps were rapidly acquired in the liver using MI‐SAGE, offering simultaneous T_1_, T_2_, and T_2_* estimates in a single breath‐held or respiratory triggered scan with strong to very strong repeatability and reproducibility.
